# The Association between Plasma Levels of Intact and Cleaved uPAR Levels and the Risk of Biochemical Recurrence after Radical Prostatectomy for Prostate Cancer

**DOI:** 10.3390/diagnostics10110877

**Published:** 2020-10-28

**Authors:** Hein Vincent Stroomberg, Gitte Kristensen, Kasper Drimer Berg, Solvej Lippert, Klaus Brasso, Martin Andreas Røder

**Affiliations:** Department of Urology, Copenhagen Prostate Cancer Center, Department of Urology, Rigshospitalet, University of Copenhagen, 2100 Copenhagen, Denmark; gittekristensen01@gmail.com (G.K.); kasperdrimerberg@gmail.com (K.D.B.); solvejlippert@gmail.com (S.L.); klaus.brasso@regionh.dk (K.B.); andreasroder@gmail.com (M.A.R.)

**Keywords:** prostate cancer, urokinase plasminogen activator receptor, biochemical recurrence, radical prostatectomy

## Abstract

Radical prostatectomy (RP) is a curatively intended treatment option for clinically localized non-metastatic prostate cancer (PCa). Novel biomarkers could refine treatment choice based on a better identification of men at risk of biochemical recurrence (BCR) following therapy. The urokinase plasminogen activator receptor (uPAR) system is a promising biomarker of aggressiveness in many cancers. The predictive value of uPAR after curatively intended treatment for PCa remains to be elucidated. This was a prospective evaluation of uPAR analysis in men with prostate cancer (Copenhagen uPAR prostate cancer (CuPCA) database). Risk of BCR following RP was analyzed using cumulative incidences with competing risk and tested with Gray’s test. Associations between quartile groups of uPAR levels and BCR were assessed with uni- and multivariate Cox proportional hazards. In total, 532 men were included. With more advanced tumor stage, Gleason score (GS), and prostate-specific antigen (PSA) the uPAR I–III + II–III plasma levels increased. Quartile levels of plasma uPAR I–III, I–III + II–III showed no significant association between the risk of BCR and the plasma uPAR levels in uni- and multivariate analysis. Despite increased levels of uPAR I–III + II–III in advanced tumor stage, intact and cleaved uPAR levels were not associated with BCR and are not predictive biomarkers for BCR following curatively intended treatment of PCa. It is unlikely that further studies of uPAR in RP treated patients is needed.

## 1. Introduction

Curatively intended treatment of clinically localized or locally advanced non-metastatic prostate cancer (PCa) includes several possible treatments whereof radical prostatectomy (RP) and external beam radiation therapy (EBRT) are considered standard treatment options. Treatment choice is based on tumor characteristics, patient-related factors including comorbidity, and patient preference [1]. A consecutive rise in prostate-specific antigen (PSA) above 0.2 ng/mL following RP is considered as biochemical recurrence (BCR) and as a relapse of the disease. On average, 25% of men undergoing curatively intended treatment will experience BCR and the risk of BCR is closely associated with tumor characteristics at baseline [2,3]. Several biomarkers in tissue and blood are being investigated but none has yet made it into clinical practice, except for PSA, and no prediction model has been able to predict the risk of BCR with 100% specificity. New biomarkers could be a mean to a more accurate identification of men at the highest risk of BCR at baseline. Based on this identification, men could be directed to specific adjuvant treatments or more intense follow-up programs. The urokinase plasminogen activator receptor (uPAR) system has long been considered a promising biomarker of aggressiveness in many cancers [4]. Like PSA, uPAR is measured in the blood and could be easily implemented in every-day practice.

The glycoprotein uPAR consists of three domains that are anchored to the cell surface by a glycolipid attached to domain III. Through allocating urokinase plasminogen activator (uPA) to the cell surface, uPAR plays a vital role in pericellular proteolysis [5]. uPAR is cleaved by uPA into two domains with domain I released in the blood and domains II and III in complex attached to the cell. Other phospholipases can cleave the glycolipid releasing both full-length uPAR (domain I–III) and the cleaved uPAR (domain II–III) in the blood as well. Three forms (I–III, I–III + II–III and I) can be measured separately in the blood, with the cleaved forms indicating an active uPA system [5,6,7]. Up-regulation of uPAR occurs during stress, injury, and inflammation of the tissue, whereas the expression is low in normal tissue [8,9,10]. A limitation of previous studies is that it is impossible to distinguish between the cleaved and intact uPAR in tissue analyses. The different cleaved forms of uPAR may hold individual predictive ability with high levels of cleaved uPAR being associated with cancer prognosis in several cancer types including PCa [11,12]. The predictive value of the three uPAR forms in terms of risk of BCR following curatively intended treatment remains to be elucidated. Therefore, we aimed to define the value of the uPAR forms in predicting BCR following RP for PCa.

## 2. Materials and Methods

### 2.1. CuPCa Database

Patient samples from the Copenhagen uPAR prostate cancer (CuPCa) database were included in this evaluation. The CuPCa database is a prospective database that has been ethically approved by the Danish National Committee on Biomedical Research Ethics for the Capital Region (no.: H-4-2011-071). The database holds information on D’Amico risk stratification, pathological tumor stage (pT-category), tumor size, lymph node status, metastatic status, Gleason score (GS), and PSA [13,14]. In short, all men referred to the Department of Urology at Rigshospitalet in Denmark for diagnostic work-up and treatment of PCa from 1 February 2012 to 1 February 2015 were included after written informed consent. At diagnosis and three months after diagnosis, blood samples were taken by vein puncture, after which uPAR levels were analyzed with time-resolved immunofluorescence assay. The database and uPAR analysis have been previously described in detail [14]. Men were excluded from the analysis if recurrence status was missing, uPAR levels were measured after treatment or not measured, and if any of the variables used for multivariate adjustment was missing. No exclusion was made for adjuvant radio therapy (RT).

### 2.2. Statistics

uPAR levels at diagnosis were statistically assessed for normality both graphically and with the Shapiro-Wilk test. In this study differences in uPAR levels at diagnosis were assessed by either parametric analysis of variance (ANOVA) with post-hoc Welch two-sample T-test or non-parametric by Kruskal-Wallis rank-sum test with post-hoc Wilcoxon rank-sum test. Descriptive analyses of continuous variables were reported with median and interquartile range (IQR) and categorical values in numbers and concurrent percentages. For time dependent analysis men were categorized based on the quartile ranges of the measured uPAR levels. Risk of BCR was analyzed using cumulative incidences with competing risk and tested with Gray’s test. BCR was defined as PSA ≥ 0.2 ng/mL following RP, persistent PSA after RP was considered as BCR. The underlining timescale was the time of initial treatment to an BCR, death before an BCR, or end of follow-up (3 March 2020), with BCR and death before BCR as competing events. Median follow-up time was calculated by reverse Kaplan–Meier estimate. Associations between quartile groups of uPAR levels and BCR were assessed with univariate cause-specific Cox proportional hazards and multivariate cause-specific Cox proportional hazards, associations are reported as Hazard Ratios (HR) with 95% confidence intervals (95%CI). Besides uPAR levels, the multivariate analysis contained age at start of treatment, PSA, pathological GS, margin status, pT-category, and lymph node status for men undergoing RP. Spline curves were created based on the univariate cox proportional hazard model with knots at the minimum, quartiles, mean, and maximum values of the measured uPAR. For all men who had RP and a second uPAR measurement after RP we assessed if a 10% difference in uPAR levels at diagnosis to post-surgical levels predicted BCR by grouping the men in 10% decrease of uPAR, equal uPAR and 10% increase of uPAR after RP. The models were tested for assumptions graphically for all variables with Schoenfeld residual plots for proportionality. A threshold for statistical significance was set at *p* = 0.05. All statistical analyses were performed using R v3.4.1 (R Development Core Team, Vienna, Austria). 

## 3. Results

### 3.1. Overall Characteristics

**Figure 1:**
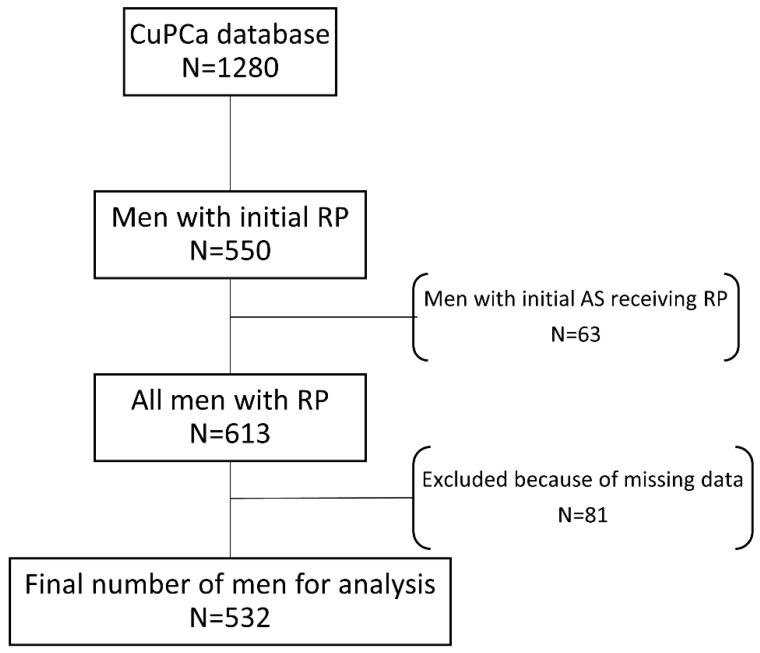
Inclusion overview of men analyzed from the Copenhagen uPAR prostate cancer (CuPCa) database. All men with radical prostatectomy (RP) as initial treatment were included. Men who got active surveillance (AS) before getting RP are also included.

A total of 550 men undergoing initial RP without neoadjuvant- or adjuvant hormone therapy were identified in the CuPCa database, patient flow is outlined in Figure 1. Following the exclusion, a total of 532 men were included in the analysis. Patient characteristics are presented in Table 1. Cumulative incidence of BCR after RP was 27.7% (95%CI: 21.1–33.2).

### 3.2. Correlation of uPAR with Disease-Specific Characteristics

**Figure 2:**
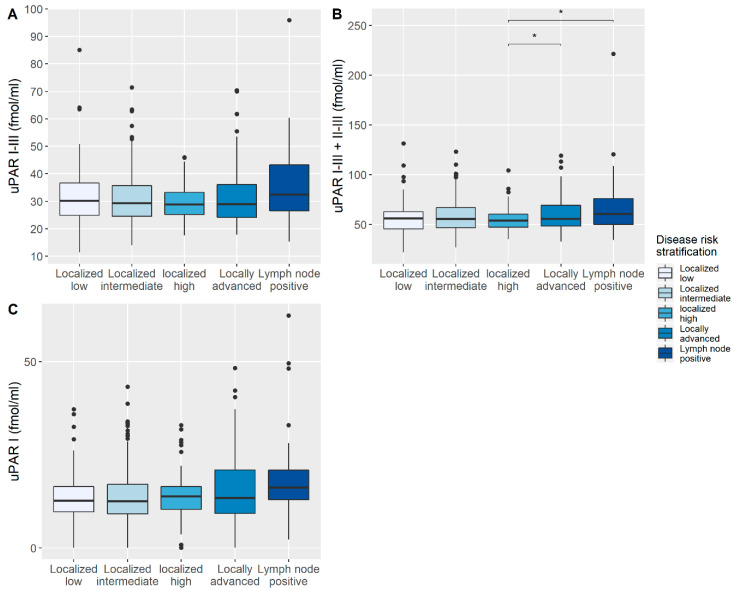
Distribution of plasma urokinase plasminogen activator receptor (uPAR) levels for disease risk stratification. (**A**) plasma uPAR I-III measurements, (**B**) plasma uPAR I–III + II–III measurements and (**C**) plasma uPAR I measurements. Box center represents the median, the box edges the interquartile range (IQR) and the whiskers are drawn until the last remaining value within 1.5 * IQR, dots are individual measurements outside this range. Significance was determined by ANOVA with post-hoc Welch two sample T-test for A and B and by Kruskal-Wallis rank sum test with post-hoc Wilcoxon rank sum test for C with * *p* ≤ 0.05.

uPAR I–III and uPAR I–III + II–III were normally distributed whereas uPAR I was not. The plasma uPAR I–III + II–III levels increased significantly with increased risk category at baseline (*p* = 0.002), with localized disease having the lowest plasma uPAR I–III + II–III levels and lymph node positive disease having the highest plasma uPAR I–III + II–III levels (Figure 2).

### 3.3. Risk of Biochemical Recurrence after Radical Prostatectomy

**Figure 3:**
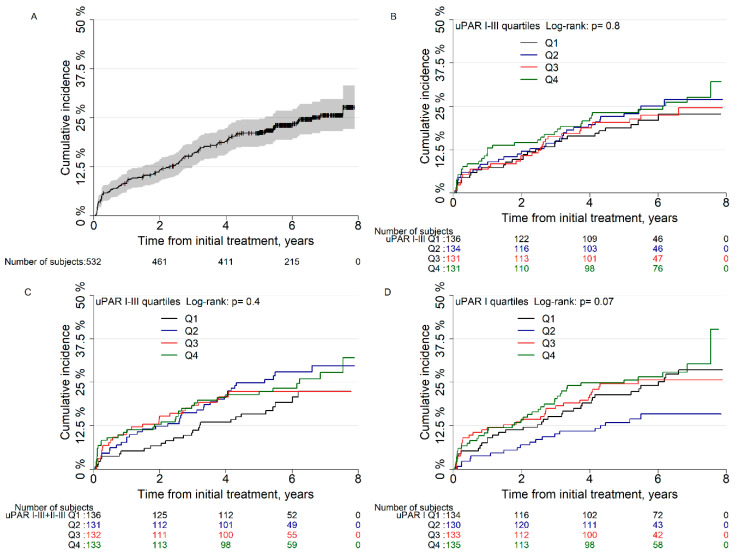
Cumulative incidence of biochemical recurrence after radical prostatectomy in (**A**) all men, (**B**) men stratified for quartiles (Q) urokinase plasminogen activator receptor (uPAR) I–III, (**C**) men stratified for quartiles uPAR I–III + II–III and (**D**) men stratified for quartiles uPAR I. Note that y axis show up to 50% cumulative incidence.

Age, PSA, pathological GS, margin status, pT-category, and lymph node status were all found to be associated with the risk of BCR in univariate analyses (Table S1). When stratified for quartile levels of plasma uPAR I–III, I–III + II–III, and I there was no difference between the groups in cumulative incidence of BCR after RP (*p* = 0.8, *p* = 0.4, and *p* = 0.07, Figure 3). In univariate analysis, the second quartile group of plasma uPAR I levels was associated with a significantly lower risk of BCR than the lowest quartile (HR: 0.56, 95%CI: 0.32–0.98, *p* = 0.04), but in multivariate analysis no significance was shown (*p* = 0.2). No further associations between plasma uPAR levels and risk of BCR were found (Table 2). Spline curves of the different plasma uPAR levels showed that there was no clear association between any plasma levels of uPAR and risk of BCR after RP (Supplementary Figure S1). Postoperative plasma uPAR measurements were available in 249 men (Supplementary Table S2). Changes in uPAR postoperatively were not significantly correlated to the risk of BCR (Supplementary Table S3).

## 4. Discussion

Despite a significant research activity and several promising biomarkers that have been found associated with BCR, none have been implemented into clinical practice. This indicates that biomarkers do not add additional information for risk assessment. Here, we found that intact and cleaved plasma uPAR levels were increased significantly in locally advanced or lymph node positive disease. This builds on the previous findings within the CuPCa database, showing that the uPAR system is more active in men with larger tumor burden [15]. We hypothesized that high levels of cleaved uPAR could have biomarker potential in localized PCa. Although univariate analysis indicated an association with BCR this was not demonstrated in multivariate analysis. Our results point to the fact that the level of cleaved uPAR at diagnosis has no value as a biomarker in localized PCa [14]. Especially because, plasma uPAR levels, intact or cleaved, were not associated with BCR after curatively intended treatment. It is therefore unlikely that uPAR yields clinical application as a biomarker in patients treated with RP.

The interest for uPAR levels in PCa men was established several years ago, but over time results have been conflicting. Two previous studies looking at risk of disease recurrence after RP and plasma uPAR showed no associations [12,16]. However, Shariat et al. found in univariate analysis that the first compared to the third tertile of intact plasma uPAR was predictive for recurrence but the prognostic value disappeared in multivariate analysis [12]. Furthermore, in metastatic PCa cleaved and intact plasma uPAR have been associated with risk of overall mortality [17]. Our studies are in concordance with the previous research on men undergoing RP but did not show a similar effect in low vs. high tertile plasma uPAR, albeit more events, a longer follow up, more men, and both cleaved and intact uPAR measured (Supplementary Table S5). The difference in prognostic value of plasma uPAR between localized and metastatic PCa can be explained by the influence of the uPAR system in degrading the extracellular matrix and involvement in cell proliferation [10]. By facilitating metastatic spread, the uPAR system could be the driving force behind disease progression. In localized disease, these mechanisms may be of lesser importance or not dominant yet. According to our results the uPAR system is unlikely to be associated with disease recurrence, which is supported by added analysis in EBRT treated patients. in EBRT treated patients uPAR levels were not associated with BCR either (Supplementary Table S7, Supplementary Figures S2 and S3). Caution needs to be taken with interpreting the EBRT results as median follow up was 6.4 years and most patients received adjuvant hormone therapy for three years. The uPAR system is often considered a marker for aggressive disease, and men allocated to undergo curatively intended treatment are selected based on their favorable risk stratification and may not have a tumor burden leading to highly elevated uPAR levels. A previous study from our group has demonstrated, that PCa men undergoing RP have increased survival, even compared to a background population without cancer [1,18]. This is also seen in the lower uPAR levels of our cohort compared to the metastatic PCa cohort [17]. Therefore, we analyzed the risk of BCR depending on the change in plasma uPAR following RP. In theory, persisting or increasing plasma uPAR levels following RP could be indicative of aggressive disease, but our analyses showed no association with uPAR levels and BCR after RP. Another addition was the subgroup analysis for patients with increased risk of recurrence based on D’Amico risk stratification. In this sub analysis no associations between uPAR levels and BCR were found (Supplementary Table S4). Our results, taken together with previous research, show that is unlikely that uPAR will have a clinical application as predictive marker for men treated with RP.

In other cancers, there has been a great interest in uPAR levels as a marker for cancer survival. This includes breast cancer, which share many similarities with PCa. In breast cancer, it was initially shown that cellular uPAR was predictive for overall survival but not relapse-free survival [19]. A later study suggested that serum uPAR is predictive for progression-free survival and overall survival in breast cancer, but the patient group was highly varied including both aggressive and non-aggressive breast cancer patients [11]. In bladder cancer, a Danish study showed no association between uPAR staining in tissue and survival following cystectomy [20]. Furthermore, preoperative plasma uPAR was not associated with disease outcome after cystectomy [21]. In contrast to these results, the uPAR system has been demonstrated to have a significant impact on survival in colorectal cancer, small cell lung cancer, and non-small cell lung cancer [22,23,24,25,26]. In lung cancer, a meta-analysis has shown a significant association between uPAR and overall survival, but this result was mainly driven by the Danish studies, whereas studies from other countries could not establish the same association [24]. A big problem with the meta-analysis was that no distinction was made between tissue and plasma uPAR. Moreover, association between plasma uPAR and disease outcome in colorectal cancer was most pronounced in more invasive forms of the disease [25]. Thus, most data so far support the hypothesis that the uPAR system mainly holds predictive value in aggressive forms of cancer. As the uPAR system could be a marker for any type of inflammation, increasing risk of death, raises the question of whether it is not solely a marker for deteriorating general health. This could be elucidated by identifying the men in which the uPAR system is upregulated in the tumor beforehand with genetic analysis. 

In this study, we prospectively evaluated uPAR levels in a consecutive cohort of men referred for diagnostic work-up and treatment of PCa. In contrary to previous research, we have assessed all isoforms of uPAR which is a significant strength of the analysis. To the best of our knowledge, our database contains the largest cohort of uPAR levels in localized PCa patients to date. Patient management followed the same guidelines throughout the study period, and level of uPAR did not influence clinical decisions. Only a limited number of men had missing data or were lost to follow-up. However, there were limitations worth mentioning. Although men with different initial treatments as are present in the database, the follow-up time is for now too limited to analyze these men. Also, analysis of subsequent treatment was not possible because of limited events and thus subsequent treatments are only shown descriptively (Supplementary Table S6). Furthermore, the overall limited follow-up did not allow for investigation of other endpoints such as time to metastasis and death.

## 5. Conclusions

In this study, we could not establish uPAR levels as a predictive biomarker for BCR following RP for localized PCa. Taken together with other studies, it is unlikely that further studies of uPAR in early-stage PCa will demonstrate to hold prognostic information beyond already established parameters. It remains uncertain if uPAR has a role as a prognostic biomarker in advanced disease. 

## Figures and Tables

**Table 1:** Overall patient characteristics for patients receiving radical prostatectomy for prostate cancer. Abbreviations: IQR: Interquartile range; PSA: Prostate-specific antigen; uPAR: Urokinase plasminogen activator receptor.

Variable		Radical Prostatectomy (N = 532)
uPAR (I–III) fmol/mL, median [IQR]		29.3 [24.7–35.6]
uPAR (I–III + II–III) fmol/mL, median [IQR]		55.6 [46.8–65.1]
uPAR (I) fmol/mL, median [IQR]		13.2 [9.7–17.2]
Follow-up time (years), median [IQR]		6.2 [5.3–7.1]
Age (years), median [IQR]		64.6 [59.5–67.9]
PSA (ng/mL), median [IQR]		8.0 [5.9–12.0]
Gleason score (%)	≤6	145 (27.3)
	3 + 4	246 (46.2)
	4 + 3	89 (16.7)
	≥8	52 (9.8)
Margin status (%)	Negative	416 (78.2)
	Positive	116 (21.8)
Tumor stage (%)	T2a + b	61 (11.5)
	T2c	340 (63.9)
	T3	131 (24.6)
Lymph node status (%)	Negative	502 (94.4)
	Positive	30 (5.6)

**Table 2:** Uni- and multivariate analysis of biochemical recurrence after radical prostatectomy (N = 532) stratified for plasma urokinase plasminogen activator receptor (uPAR) quartiles. Multivariate analysis was adjusted for age at start of treatment, prostate-specific antigen, pathological Gleason score, margin status, pathological tumor stage and lymph node status. Abbreviations: HR: Hazard ratio; 95%CI: 95% confidence interval; Q: Quartile. * Statistically significant as defined by *p*-Value below 0.05.

		Univariate Analysis		Multivariate Analysis	
		HR (95%CI)	*p*-Value	HR (95%CI)	*p*-Value
uPAR I-III	Q1	Reference		Reference	
	Q2	1.20 (0.73–1.99)	0.47	1.16 (0.70–1.94)	0.56
	Q3	1.07 (0.64–1.80)	0.80	1.26 (0.74–2.15)	0.40
	Q4	1.26 (0.76–2.07)	0.37	1.32 (0.79–2.18)	0.29
uPAR I-III + II-III	Q1	Reference		Reference	
	Q2	1.49 (0.90–2.47)	0.12	1.32 (0.79–2.33)	0.29
	Q3	1.19 (0.70–2.03)	0.52	1.35 (0.78–2.15)	0.28
	Q4	1.38 (0.83–2.31)	0.21	1.27 (0.75–2.15)	0.38
uPAR I	Q1	Reference		Reference	
	Q2	0.56 (0.32–0.98)	0.04 *	0.68 (0.38–1.22)	0.20
	Q3	1.01 (0.63–1.63)	0.95	1.08 (0.66–1.75)	0.76
	Q4	1.14 (0.72–1.81)	0.58	1.01 (0.63–1.62)	0.96

## Supplementary Materials

The following are available online at https://www.mdpi.com/2075-4418/10/11/877/s1, Figure S1: Spline curves of hazards of biochemical recurrence after radical prostatectomy for plasma uPAR levels at baseline, Figure S2: Cumulative incidence of biochemical recurrence after external beam radiation therapy in A) all men, B) men stratified for quartiles uPAR I-III, C) men stratified for quartiles uPAR I-III + II-III and D) men stratified for quartiles uPAR I, Figure S3: Spline curves of hazards of biochemical recurrence after external beam radiation therapy for plasma uPAR levels at baseline, Table S1: Univariate analysis for all adjusted variables in multivariate analysis, Table S2: Patient characteristics for men with second plasma uPAR measurement, Table S3: Univariate analysis of change in plasma uPAR after radical prostatectomy and biochemical recurrence, Table S4: Subgroup analysis of plasma uPAR levels based on D’Amico categorization, Table S5: Median and tertile analysis of plasma uPAR, Table S6: subsequent treatment for men with biochemical recurrence (N = 125), Table S7: Univariate analysis of biochemical recurrence after external beam radiation therapy (N = 159) stratified for plasma uPAR quartiles, Text S1: Detailed number of excluded men based on exclusion criteria.
