# Supplementary material for: The Association between Plasma Levels of Intact and Cleaved uPAR Levels and the Risk of Biochemical Recurrence after Radical Prostatectomy for Prostate Cancer
Source: Diagnostics (Basel). 2020 Oct 28;10(11):877. doi: 10.3390/diagnostics10110877 (PMC7694049; doi:10.3390/diagnostics10110877)
Supplement: Supplementary file 1 [file diagnostics-10-00877-s001.pdf]

Supplementary table 1. Univariate analysis for all adjusted variables in multivariate analysis

		Radical prostatectomy	
		HR (CI95)	P-value
Age		1.06 (1.03 – 1.1)	<0.001
PSA (log2)		1.87 (1.53 – 2.29)	<0.001
Gleason Score	≤6	Reference	
	7	4.41 (2.22 – 8.79)	<0.001
	≥8	20.76 (9.94 – 43.33)	<0.001
Margin status	Negative	Reference	
	Positive	4.52 (3.18 – 6.42)	<0.001
Pathological tumor stage	T2a+b	Reference	
	T2c	2.09 (0.75 – 5.81)	0.16
	T3	13.59 (4.97 – 37.19)	<0.001
Lymph node status	Negative	Reference	
	Positive	6.33 (3.94 – 10.16)	<0.001

Supplementary table 2. Patient characteristics for men with second plasma uPAR measurement

Variable		Radical prostatectomy (N=249)
Number of events (%)		62 (24.2)
Follow-up time (years), median [IQR]		6.0 [5.4 – 7.0]
Age (years), median [IQR]		64.4 [59.3 – 67.8]
PSA (ng/ml), median [IQR]		8.0 [6.0 – 11.0]
Gleason score (%)	≤6	62 (24.9)
	3+4	121 (48.6)
	4+3	43 (17.3)
	≥8	23 (9.2)
Margin status (%)	Negative	195 (78.3)
	Positive	54 (21.7)
Pathological tumor stage (%)	T2a+b	30 (12.0)
	T2c	165 (66.3)
	T3	54 (21.7)
Lymph node status (%)	Negative	237 (95.2)
	Positive	12 (4.8)

Supplementary table 3. Univariate analysis of change of plasma uPAR after radical prostatectomy and risk of biochemical recurrence.

		HR (CI95)	P-value
uPAR I-III	Decrease of >10%	Reference	
	Within 10% of baseline	1.67 (0.94 – 2.96)	0.08
	Increase of >10%	1.24 (0.64 – 2.37)	0.52
uPAR I-III + II-III	Decrease of >10%	Reference	
	Within 10% of baseline	1.04 (0.57 – 1.86)	0.90
	Increase of >10%	1.09 (0.57 – 2.07)	0.80
uPAR I	Decrease of >10%	Reference	
	Within 10% of baseline	1.20 (0.57 – 2.54)	0.63
	Increase of >10%	1.46 (0.81 – 2.65)	0.21

Supplementary Table 4. Subgroup analysis of plasma uPAR levels based on D'Amico categorization.

		Localized low risk (N=131)		Localized intermediate risk (N=204)		Localized high risk (N= 75)	
		HR (CI95)	P-value	HR (CI95)	P-value	HR (CI95)	P-Value
uPAR I-III	Q1	Reference		Reference		Reference	
	Q2	1.06 (0.15 – 7.57)	0.95	2.54 (0.78 – 4.23)	0.12	0.94 (0.25 – 3.49)	0.92
	Q3	2.11 (0.38 – 11.54)	0.39	1.43 (0.38 – 5.33)	0.60	1.54 (0.46 – 5.11)	0.48
	Q4	0.83 (0.12 – 5.90)	0.85	1.35 (0.38 – 4.81)	0.65	1.69 (0.42 – 6.77)	0.4+
uPAR I-III+II-III	Q1	Reference		Reference		Reference	
	Q2	2.53 (0.42 – 15.17)	0.31	1.21 (0.37 – 3.96)	0.76	0.62 (0.19 – 2.03)	0.43
	Q3	2.03 (0.37 – 11.08)	0.41	1.45 (0.44 – 4.75)	0.54	1.22 (0.42 – 3.52)	0.71
	Q4	0.69 (0.05 – 6.37)	0.77	1.22 (0.39 – 3.86)	0.73	0.50 (0.10 – 2.46)	0.39
uPAR I	Q1	Reference		Reference		Reference	
	Q2	1.45 (0.24 – 8.70)	0.68	0.36 (0.10 – 1.32)	0.12	1.09 (0.29 – 4.11)	0.90
	Q3	2.03 (0.37 – 11.06)	0.42	0.75 (0.25 – 2.24)	0.60	1.52 (0.47 – 4.97)	0.49
	Q4	0.58 (0.05 -6.37)	0.65	0.90 (0.33 – 2.42)	0.83	0.74 (0.16 – 3.30)	0.69

Supplementary Table 5. Median and tertile analysis of plasma uPAR.

		HR (CI95)	P-value		HR (CI95)	P-Value
uPAR I-III	Below median	Reference		T1	Reference	
	Above median	1.06 (0.74 – 1.50)	0.75	T2	1.22 (0.79 – 1.88)	0.37
				T3	1.07 (0.69 – 1.67)	0.76
uPAR I-III+II-III	Below median	Reference		T1	Reference	
	Above median	1.04 (0.73 – 1.48)	0.82	T2	1.04 (0.67 – 1.61)	0.87
				T3	1.23 (0.80 – 1.89)	0.34
uPAR I	Below median	Reference		T1	Reference	
	Above median	1.38 (0.97 – 1.96)	0.08	T2	1.03 (0.66 – 1.61)	0.90
				T3	1.32 (0.86 – 2.01)	0.20

Supplementary table S6. Subsequent treatment for men with biochemical recurrence (N=125)

Type of subsequent treatment	N (%)
External beam radio therapy (EBRT)	48 (38.4)
Bicalutamide	26 (20.8)
Androgen depletion therapy (ADT)	5 (4.0)
Dendrite cell therapy	1 (0.8)
No second treatment recorded	45 (36.0)

Supplementary Table S7. Univariate analysis of biochemical recurrence after external beam radiation therapy (N=159) stratified for plasma uPAR quartiles.

		Univariate analysis	
		HR (CI95)	P-value
uPAR I-III	Q1	Reference	
	Q2	2.92 (0.56 – 15.01)	0.20
	Q3	2.08 (0.38 – 11.35)	0.40
	Q4	0.95 (1.05 – 6.77)	0.96
uPAR I-III + II-III	Q1	Reference	
	Q2	0.19 (0.02 – 1.60)	0.13
	Q3	0.58 (0.14 – 2.41)	0.45
	Q4	0.77 (0.21 – 2.88)	0.70
uPAR I	Q1	Reference	
	Q2	2.64 (0.48 – 14.51)	0.27
	Q3	0.58 (0.58 – 15.54)	0.19
	Q4	1.03 (0.15 – 7.33)	0.98

Supplementary figure 1. Spline curves hazards of biochemical recurrence after radical prostatectomy for plasma uPAR levels (fmol/ml) at baseline. A) plasma uPAR I-III, B) plasma uPAR I-III + II-III and C) plasma uPAR I.

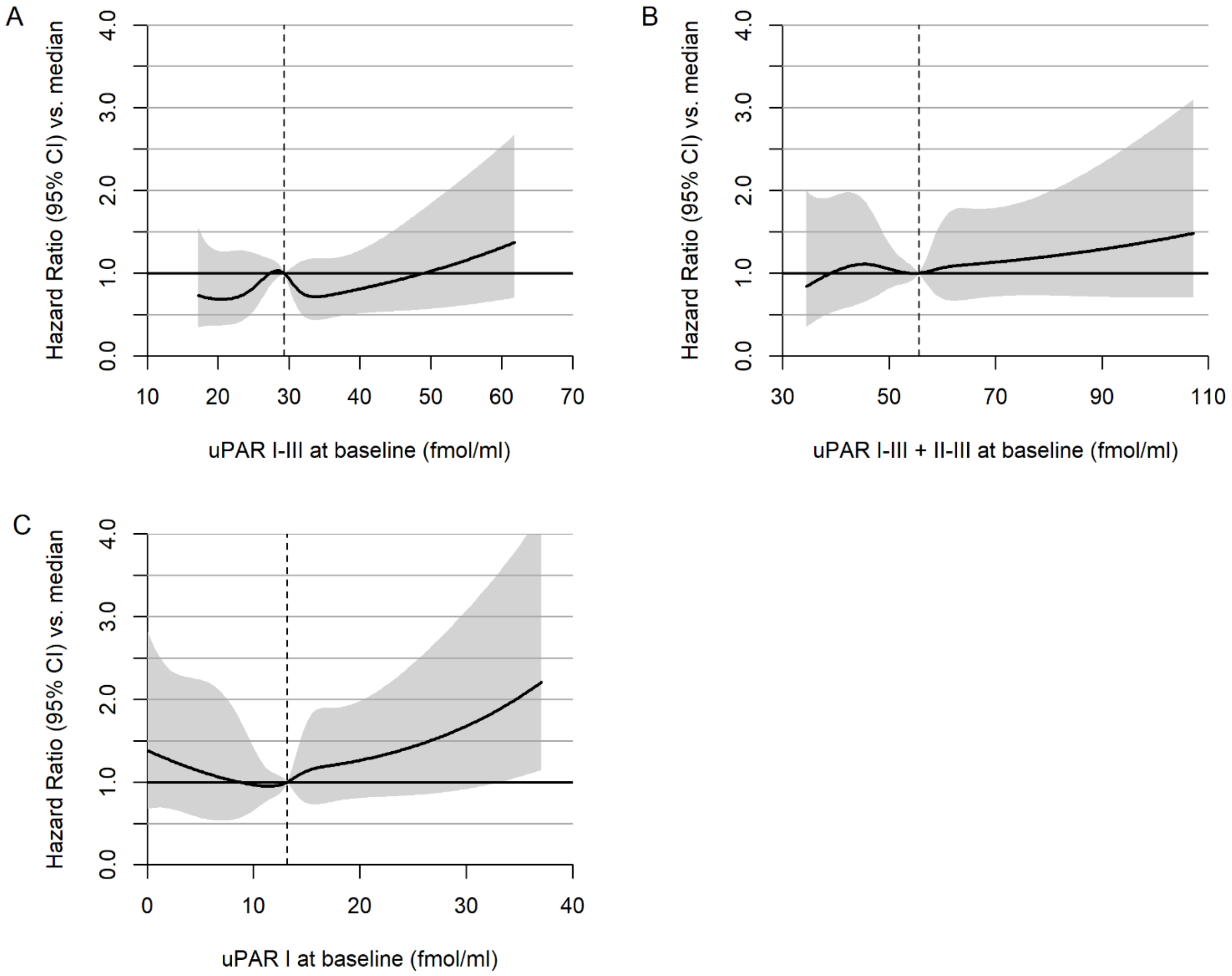

Supplementary figure 2. Cumulative incidence of biochemical recurrence after external beam radiation therapy in A) all men, B) men stratified for quartiles uPAR I-III, C) men stratified for quartiles uPAR I-III + II-III and D) men stratified for quartiles uPAR I.

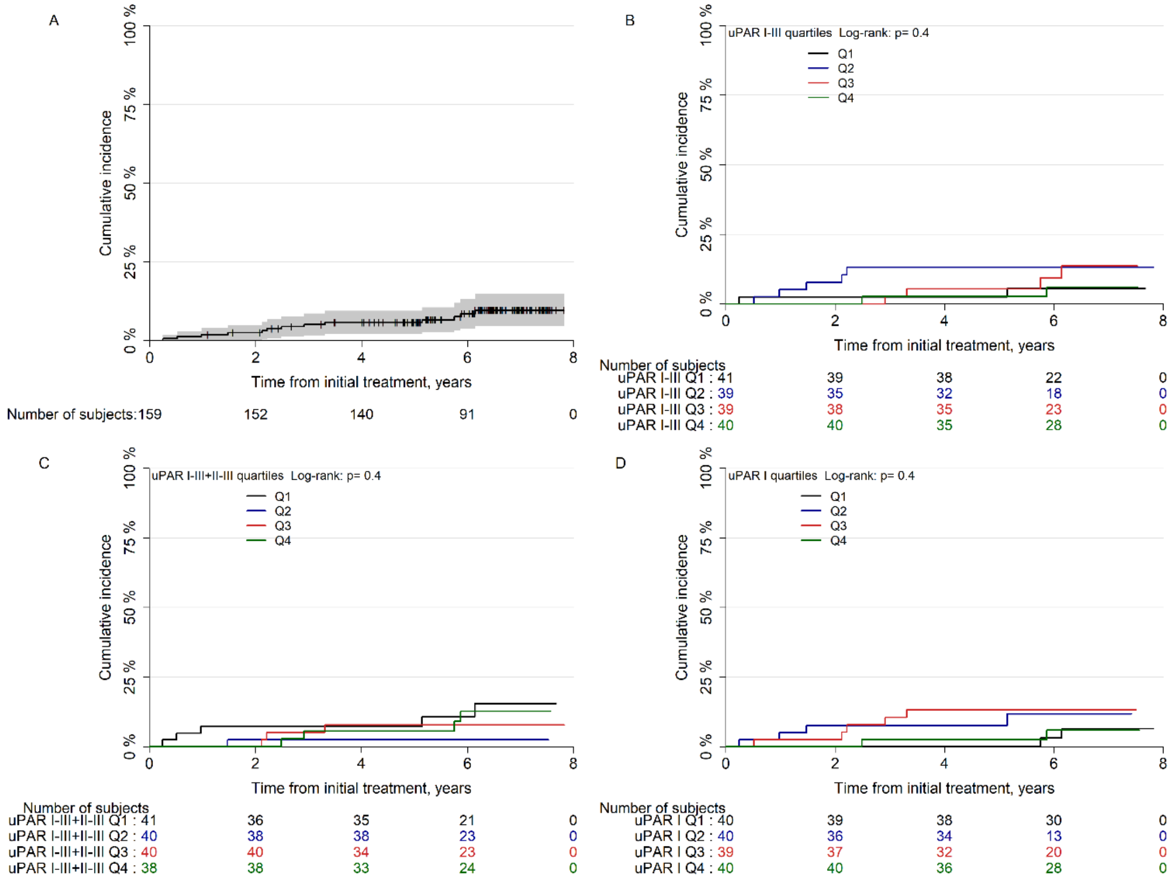

Supplementary figure 3. Spline curves hazards of biochemical recurrence after external beam radiation therapy for different plasma uPAR levels (fmol/ml) at baseline. A) plasma uPAR I-III, B) plasma uPAR I-III + II-III and C) plasma uPAR I.

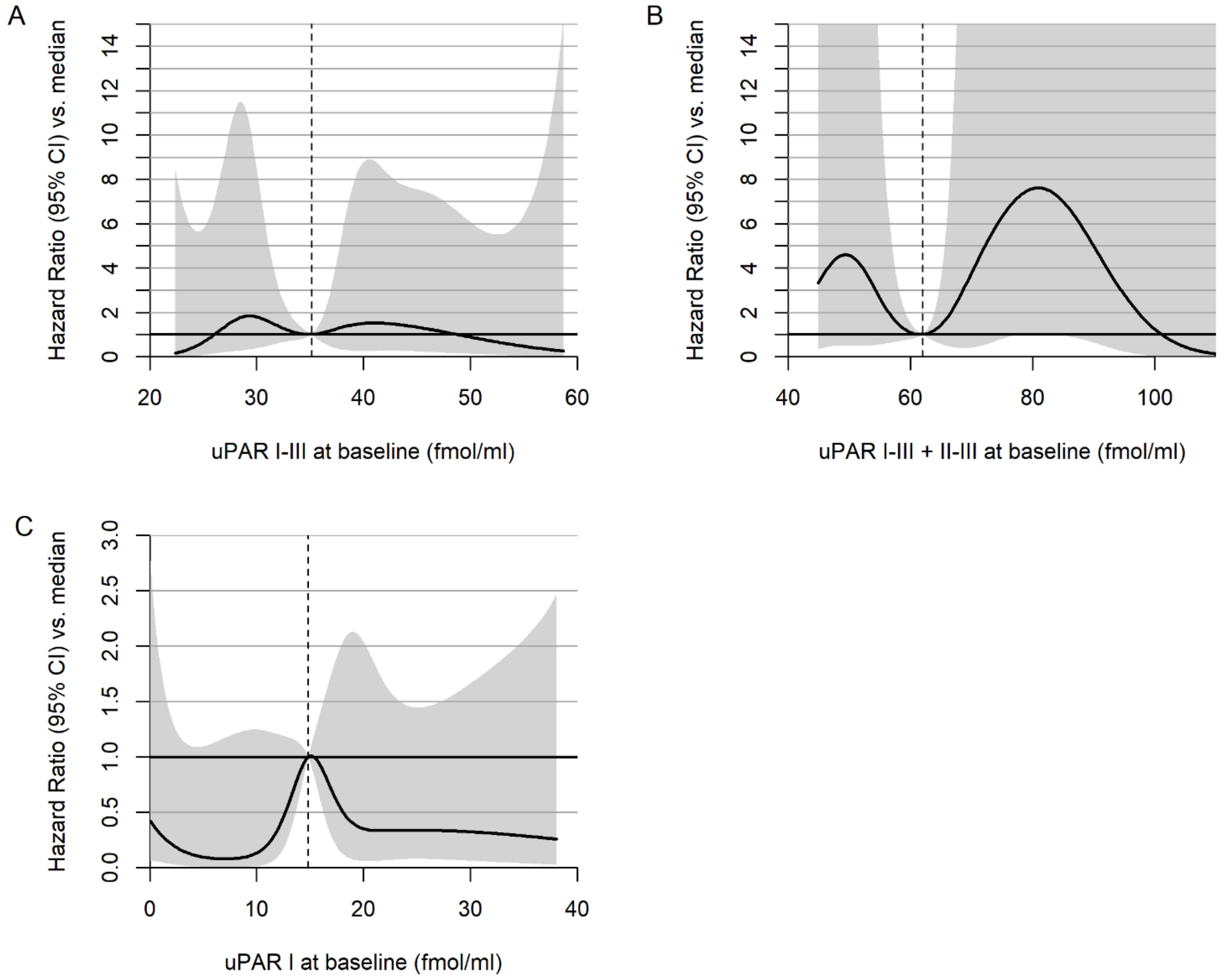

Supplementary text S1. Detailed number of excluded men based in exclusion criteria.

Exclusion criteria:

Missing recurrence status, uPAR must be measured before treatment, uPAR has to have been measured, all variables that are adjusted for in multivariate analysis must be present.

Breakdown of exclusion per curative treatment

For Radical Prostatectomy

Initially 613 patients

-1 patient for missing recurrence status

-8 patient for having the uPAR measured after the treatment

-58 patient for missing uPAR measurements

-14 patient for missing pretreatment PSA or margin status

For external beam radio therapy

Initially 203 patients

-5 patient for missing recurrence status

-9 patient for having uPAR measured after the treatment

-25 patient for missing uPAR measurements

-5 patient for missing pretreatment PSA
